# Retinitis pigmentosa-linked mutations impair the snRNA unwinding activity of SNRNP200 and reduce pre-mRNA binding of PRPF8

**DOI:** 10.1007/s00018-025-05621-z

**Published:** 2025-03-05

**Authors:** Felix Zimmann, Francois McNicoll, Prasoon Kumar Thakur, Michaela Blažíková, Jan Kubovčiak, María Clara Hernández Cañás, Zora Nováková, Cyril Bařinka, Michal Kolář, David Staněk, Michaela Müller-McNicoll, Zuzana Cvačková

**Affiliations:** 1 Institute of Molecular Genetics, Czech Academy of Sciences, Prague, Czech Republic; 2 Institute of Molecular Biosciences, Goethe University, Frankfurt, Germany; 3 Institute of Biotechnology, Czech Academy of Sciences, Vestec, Czech Republic; 4 Max Planck Institute of Biophysics, Frankfurt, Germany

**Keywords:** iCLIP, Pre-mRNA splicing, PRPF8, SNRNP200, Retinitis pigmentosa

## Abstract

Retinitis pigmentosa (RP) is a hereditary disorder caused by mutations in more than 70 different genes including those that encode proteins important for pre-mRNA splicing. Most RP-associated mutations in splicing factors reduce either their expression, stability or incorporation into functional splicing complexes. However, we have previously shown that two RP mutations in PRPF8 (F2314L and Y2334N) and two in SNRNP200 (S1087L and R1090L) behaved differently, and it was still unclear how these mutations affect the functions of both proteins. To investigate this in the context of functional spliceosomes, we used iCLIP in HeLa and retinal pigment epithelial (RPE) cells. We found that both mutations in the RNA helicase SNRNP200 change its interaction with U4 and U6 snRNAs. The significantly broader binding profile of mutated SNRNP200 within the U4 region upstream of the U4/U6 stem I strongly suggests that its activity to unwind snRNAs is impaired. This was confirmed by FRAP measurements and helicase activity assays comparing mutant and WT protein. The RP variants of PRPF8 did not affect snRNAs, but showed a reduced binding to pre-mRNAs, which resulted in the slower splicing of introns and altered expression of hundreds of genes in RPE cells. This suggests that changes in the expression and splicing of specific genes are the main driver of retinal degeneration in PRPF8-linked RP.

**Supplementary Information:**

The online version contains supplementary material available at 10.1007/s00018-025-05621-z.

## Introduction

Pre-mRNA splicing is a crucial processing step in eukaryotic gene expression. Most protein-coding genes contain non-coding introns, which have to be efficiently and precisely removed and the coding exons joined together to generate functional mRNAs. The process of pre-mRNA splicing comprises two sequential transesterification reactions that are catalyzed by a multicomponent complex called the spliceosome. The spliceosome is assembled on pre-mRNAs from preformed small nuclear ribonucleoprotein (snRNP) subcomplexes, each consisting of one small nuclear RNA (either U1, U2, U4, U5 or U6 snRNA) and several snRNP-specific proteins. The most complex preassembled particle is the U4/U6⋅U5 tri-snRNP, where U4 and U6 snRNAs extensively basepair and the U5 snRNP is bound via a network of protein - protein interactions [1, 2]. Formation of the catalytically active spliceosome includes a large number of specific interactions and snRNP rearrangements that ensure splicing fidelity and provide opportunities for regulation [3–6]. One of the key rearrangements during spliceosome activation is the unwinding of the U4/U6 duplex to allow U6 snRNA to create new contacts with both U2 snRNA and the pre-mRNA and to form the catalytical center of the spliceosome.

This unwinding is catalyzed by the Ski2-like RNA helicase SNRNP200 (also called BRR2), which contains two DExH-box helicase cassettes arranged in tandem. Only the N-terminal cassette possesses an RNA helicase activity while the C-terminal cassette has a regulatory function [7]. Unlike other spliceosomal helicases, SNRNP200 remains associated with its substrate before and during splicing and its activity must therefore be tightly regulated to ensure proper timing of U4/U6 unwinding and spliceosome activation. The main known regulator of SNRNP200 is PRPF8, a major, evolutionarily conserved building block of the spliceosome. PRPF8 is the largest protein of the spliceosome and contacts all pre-mRNA sequences critical for splicing: 5′ splice sites (5′ss), branch points (BP) and 3′ splice sites (3′ss), as well as spliceosomal snRNAs. Conformational changes in PRPF8 are essential for remodeling the RNA catalytic core during splicing and regulation of SNRNP200 activity [6, 8–19].

Mutations in PRPF8 and SNRNP200 are found in patients with retinitis pigmentosa (RP), a human hereditary disease characterized by the loss of photoreceptors and degenerative changes in the retinal pigment epithelium (RPE) [20–23]. In addition, mutations in PRPF8 are involved in glaucoma [24] and cancer [25]. Currently, it is unclear how mutations in ubiquitously required core components of the splicing machinery lead to cell type-specific disorders. All RP-linked mutations of PRPF8 are located in the Jab1/MPN domain, which interacts with and regulates SNRNP200’s activity. Previously, we have shown that six out of eight studied RP mutations abolished interactions with other spliceosomal proteins impairing the incorporation of PRPF8 into the U4/U6⋅U5 tri-snRNP [26]. In fact, most mutations in splicing factors have been shown to impair protein–protein interactions with other spliceosomal proteins [27–32]. As a consequence, splicing is often deregulated resulting in miss-splicing and aberrant gene expression of a vast number of genes not limited to but including those involved in retinal and neural function [33–39]. Most RP-linked mutations in PRPF8, which is an essential splicing factor, inhibit formation of the tri-snRNP, [26]. Intriguingly, two mutations that cause RP, namely F2314L and Y2334N [40, 41], do not inhibit tri-snRNP formation, but still reduced the splicing efficiency of specific gene reporters [26]. Similar observations were made for two RP-linked mutations in SNRNP200 (S1087L and R1090L), where incorporation of mutated SNRNP200 into the U4/U6⋅U5 tri-snRNP was not affected [42]. All four mutations cause retinal degeneration, but it remains unclear how these mutations affect protein functions. We hypothesize that the studied RP mutations alter the interaction of PRPF8 and SNRNP200 with RNA. So far, studies that addressed the impact of RP-linked mutations on RNA-binding were limited to in vitro studies or non-mammalian systems such as budding yeast. While these studies might provide important insights into the basic mechanism, they fall short of reflecting the spliceosomal context in mammalian cells.

Therefore we employed individual-nucleotide resolution UV crosslinking and immunoprecipitation (iCLIP) to determine the binding profiles of both studied proteins with snRNAs and pre-mRNAs and fluorescence recovery after photobleaching (FRAP) to study in cellulo dynamics of PRPF8 association with RNA. We utilized HeLa cell lines stably expressing GFP-tagged SNRNP200 or PRPF8 and compared protein-RNA interactions of WT proteins with RP variants. In addition, we established RPE cell lines expressing either PRPF8^WT^-GFP or PRPF8^Y2334N^-GFP. Our data reveal how the studied RP mutations affect interactions with RNAs and the function of the proteins within the spliceosomal context and suggest molecular mechanisms underlying retinal degeneration caused by the mutations in these splicing factors.

## Material and methods

### Cell culture

HeLa and RPE cells were cultured in high glucose (4.5 g/l) DMEM (Sigma) supplemented with 10% fetal bovine serum (Gibco) and Penicillin-Streptomycin (Gibco) at 37 °C and 5% CO_2_.

### GFP fluorescence acquisition

For GFP fluorescence detection, cells were cultured on coverslips, washed with PBS, fixed with 4% (w/v) paraformaldehyde/PIPES for 10 min and mounted with Fluoromount G (Southern Biotech) containing DAPI. Images were acquired using a DeltaVision microscope system (Applied Precision) equipped with an oil immersion PLAN APO N 63x objective (1.42 NA) and further processed by Huygens deconvolution (Huygens Professional, version 22.04).

### Protein lysate preparation and western blot analysis

Proteins were separated by 8% polyacrylamide gel containing SDS (SDS-PAGE) and transferred to nitrocellulose membranes (Protran). 5% (w/v) nonfat milk in PBST (0.05% Tween-20 in PBS) was used for blocking the membrane, primary and secondary antibodies were diluted in 2% (w/v) nonfat milk in PBST. Secondary antibodies were conjugated with horseradish peroxidase, whose activity was detected using the Super Signal West Femto Maximum Sensitivity Substrate (Thermo Scientific).

### Chromatin fraction isolation, RNA isolation, RT-qPCR

All buffers used for chromatin fraction isolation were pre-chilled on ice. Cells were scraped into PBS / 1 mM EDTA and collected by centrifugation. The pellet was lysed with NP-40 lysis buffer (10 mM Tris - HCl pH 7.5, 0.15% NP-40, 150 mM NaCl) for 5 min, placed over 2.5 volumes of sucrose cushion (24% sucrose in NP-40 lysis buffer) and centrifuged for 10 min at 4 °C. The pellet (nuclei) was rinsed with PBS / 1 mM EDTA and resuspended in glycerol buffer (20 mM Tris - HCl, pH 7.9, 75 mM NaCl, 0.5 mM EDTA, 0.85 mM DTT, 0.125 mM PMSF, 50% glycerol), an equal volume of nuclei lysis buffer (10 mM HEPES, pH 7.6, 1 mM DTT, 7.5 mM MgCl_2_, 0.2 mM EDTA, 0.3 M NaCl, 1 M UREA, 1% NP-40) was added, the tube was incubated for 2 min on ice and centrifuged. The pellet (chromatin fraction) was washed with PBS / 1 mM EDTA. RNA from the chromatin fraction pellet was isolated with TRIZOL (Invitrogen) according to the manufacturer’s protocol, with an additional phenol/chloroform extraction step prior to isopropanol precipitation. RNA was DNase treated (Turbo DNA free kit, Ambion) and cDNA was synthesized with SuperScript III RT (Invitrogen) using random hexamers. RT-qPCR was performed using the SYBR Green method (Roche) on a LightCycler 480 System (Roche). Splicing efficiency (pre-mRNA/mRNA) was calculated as 2^(Ct[mRNA]–Ct[pre-mRNA])^. Primers are listed in the supplementary table (Table S1). Plots and statistical analyses were performed using the PRIZM GraphPad software. The statistical significance of differences was evaluated with *t-*test. * *p* < 0.05.

### Recombinant protein production and purification

The N-terminal cassettes (amino acids 395-1324, according to [43]) of SNRNP200 WT and RP-mutants (S1087L, R1090L) were cloned into the pDEST MM322 destination vector featuring N-terminal Twin-Strep-FLAG-Halo tags [44] using the Gateway technology (Thermo Fisher Scientific). pMM322 SNRNP200 vectors were transiently transfected into suspension HEK293T cells using linear polyethyleneimine (Polysciences) [45]. Cells were harvested 3 days later by centrifugation at 500×g, 4 °C, 5 min, resuspended in a lysis buffer (100 mM Tris - HCl, pH 8.0, 10 mM NaCl, 5 mM KCl, 2 mM MgCl_2_, 10% glycerol) supplemented with benzonase (Millipore) and a protease inhibitor coctail (Merck) and lysed by sonication. The lysate was supplemented with 0.2% (v/v) Igepal 630 and incubated on ice for 10 min. Next NaCl was added to a final concentration of 150 mM followed by incubation for additional 10 min on ice. The lysate was centrifuged at 9000×*g*, 15 min, 4 °C and subsequently at 30,000×*g*, 30 min, 4 °C. The supernatant was filtered through a 0.45 μm filter and mixed with a StrepTactin XT 4Flow high capacity resin (IBA). Following 1 h incubation at 4 °C, the resin was washed thoroughly with the lysis buffer. Protein fusions were eluted with the lysis buffer supplemented with 10 mM D-biotin and the Strep-FLAG-Halo tag was cleaved off by the TEV protease (20:1 molar ratio) at 4 °C overnight.

### Helicase assay

The substrate for the helicase assay was designed as a linear 12 nt RNA duplex with a 31 nt overhang at the 3′ end according to [43]. The 12 nt RNA oligo (5′-CGGCUCGCGGCC-Cy5-3′) was labeled with Cy5 on the 3′ end (Integrated DNA Technologies). The complementary RNA oligonucleotide (5′-GGCCGCGAGCCGGAAATTTAATTATAAACCAGACCGTCTCCTC-3′) was prepared by in vitro transcription using T7 RNA polymerase (Megashortscript kit, Thermo Fisher Scientific). The 12 nt Cy5 labeled oligo was annealed with twofold molar excess of the unlabeled oligo and purified from 10% native PAGE.

The helicase assay was performed with 100 nM recombinant protein and 10 nM annealed RNA substrate in presence of tenfold molar excess of unlabeled 12 nt RNA oligo (5′-CGGCUCGCGGCC-3′) in a buffer containing 40 mM Tris-HCl, pH 7.4, 50 mM NaCl, 8% glycerol, 0.5 mM MgCl_2_, 100 ng/μl BSA, RNasin (1 U/μl), 1.5 mM DTT. The reaction was running at 30 °C for 40 min in 10 µl volume with/without 1 mM ATP and quenched by adding of 10 µl of stop buffer (40 mM Tris-HCl, pH 7.4, 50 mM NaCl, 25 mM EDTA, 1% SDS, 10% glycerol). The control sample was boiled at 95 °C for 5 min. Samples were separated on a native 10% PAGE and Cy5 labeled RNA was visualized on the Amersham Typhoon 5 (GE Healthcare). Intensities of bands corresponding to unwound RNAs were quantified with ImageJ. Plots and statistical analyses were performed using the PRISM GraphPad software. The statistical significance of differences was evaluated with a *t-*test.

### RNA-seq, differential gene expression and intron retention analyses

Confluent RPE cells from 100 mm diameter Petri dishes were resuspended in 1 ml of TRIZOL (Invitrogen) and total RNA was isolated using Direct-zol microprep kit (Zymo research) including DNase I treatment. Good quality RNA (RIN > 7.3) was ribodepleted and used for library preparation. Libraries were prepared using KAPA RNA HyperPrep Kit with RiboErase (Roche) and sequenced on Illumina NextSeq 500 instrument with 2× 75 bp configuration. RNA-seq reads were mapped against the human genome (version hg38) with GENCODE gene annotation using STAR (version 2.7.8a) [46] with the following parameters: STAR --runMode alignReads --outSAMattributes All --outSAMtype BAM SortedByCoordinate --outFilterMismatchNmax 999 --outFilterMultimapNmax 1 --outFilterMismatchNoverLmax 0.04. Reads were counted into exons of genes using htseq-count (version 0.13.5) [47] with default parameters and the count tables were used as input for differential gene expression analyses with DESeq2 (version 1.30.1) [48]. An adjusted *p* value <0.05 was considered significant and |log_2_ FC| > 0.58 was the criterion for differentially expressed genes. Gene Ontology (GO) enrichment analysis was performed using hypeR (version 1.7.0) [49] tested against all genes found in DESeq2 analysis. An adjusted *p* value <0.05 was considered significant and “Biological Process” categories were explored. IRFinder (version 1.3.1) [50] software was used with default parameters to identify IR events; downstream analysis was done as outlined in IRFinder manual for two or fewer replicates. An adjusted *p* value <0.05 was considered significant and |delta IR| > 0.1 was the criterion for differentially retained introns.

### iCLIP

iCLIP was performed as described in [51] with minor modifications in three independent replicates. Briefly, cells grown on 14 cm diameter Petri dishes (with more than 90% confluence) were irradiated with 150 mJ/cm^2^ UV light (254 nm). Cells were lysed in 50 mM Tris‐HCl, pH 7.5; 100 mM NaCl, 1% IGEPAL, 0.1% SDS, 0.5% sodium deoxycholate, protease inhibitor cocktail without EDTA, sonicated and RNA was partially digested with RNase I (Invitrogen). Immunoprecipitation was done with Dynabeads protein G coupled with goat anti-GFP antibody (provided by E. Geertsma, MPI-CBG, Dresden, Germany). 3′ RNA ends were dephosphorylated and then ligated with pre-adenylated DNA 3′ adapters (Integrated DNA Technologies); 5′ RNA ends were radiolabeled. RNA - protein complexes were separated on SDS-PAGE, transferred onto nitrocellulose membrane and visualized by autoradiography. RNA:protein adducts were cut from the membrane and proteins degraded with proteinase K. RNA was reverse transcribed using reverse primers containing an adapter sequence. cDNA was size-purified on a TBE-urea gel, circularized using CircLigase II kit and annealed to an oligonucleotide complementary to the BamHI site incorporated by the reverse primer adapter sequence. Circularized cDNA was cleaved with BamHI and PCR amplified with primers complementary to adapters. iCLIP samples were sequenced on an Illumina NEXTSeq 500 instrument with 75 bp single-end reads.

### Fluorescence recovery after photobleaching measurement (FRAP)

FRAP measurements were performed using a DeltaVision microscope system (Applied Precision), SoftWorx software, equipped with a live cell imaging chamber that controls temperature (37 °C), humidity and CO_2_ concentration (5%). Photobleaching was done with a 488 nm LASER within a circular spot of approximately 1.5 µm in diameter in the nucleoplasm and the fluorescence recovery was monitored using adaptive time intervals for 100 s. Obtained time lapses were analyzed using ImageJ to subtract background and correct for bleaching. FRAP curves that represent the mean of 12-22 measurements were fitted individually with the full model and bi-exponentially using MATLAB R2021b (MathWorks, Natick, MA) as described previously [52].

### iCLIP bioinformatics analysis

Barcodes were removed from all iCLIP reads using the extract command of UMI-tools [53] and adapters were trimmed from the 3′ end using Cutadapt [54]. Processed reads were uniquely mapped to the human hg19/GRCh37 genome assembly (GENCODE v30) using Bowtie software (version 1.2.3; Bowtie parameters: −v 2 −m 1) [55]. Individually mapped replicates were merged and PCR duplicates were removed using the dedup command of UMI-tools [53]. Crosslink sites (first nucleotide of the read) were extracted using the custom shell script and selected as iCLIP crosslinks. The distribution of the iCLIP crosslinks to RNA was analyzed (as a percentage of the total number of crosslinks) and mapped to open reading frames, intergenic and intronic regions, non-coding RNA (ncRNA), and untranslated regions (UTRs). The proportion of normalized iCLIP crosslink densities (iCLIP-tags divided by average length of the region) mapped to different genomic regions was calculated. Individual replicates had the same tendency as the merged sample but the merged sample allowed us to confidently identify the binding sites.

### Differential binding analysis

To obtain differential binding between WT and mutant (Mut), we extracted the ‘protein-coding gene’ biotype from GENCODE v30 annotation (gtf). Crosslink counts for protein-coding genes were determined using bedtools intersect (v 2.30.0) (with parameters: −s −c) [56] for WT and Mut samples from processed iCLIP data in bed format (described in previous section). Genes where the average number of crosslinks for the WT and Mut samples was equal to or greater than 10 were selected for further analysis (Crosslinks in WT + Crosslinks in Mut/2 ≥ 10). Crosslink counts were normalized to counts per million (CPM) using the formula: CPM = crosslink counts over the individual gene * 10^6^/total number of crosslinks in the individual sample. To determine genes differentially bound by RP variants we applied the following formula that normalized differences in WT and Mut cross-links to total crosslinks to particular mRNA region: difBinding = (Normalized crosslinks in WT − Normalized crosslinks in Mut)/(Normalized crosslinks WT + Normalized crosslinks Mut)/2. The calculations were done using custom R script. Binding intensity differences (difBinding) between WT and Mut, ≥0.5 or ≤−0.5 were considered for further analysis. Gene ontology (GO) enrichment analysis for the differentially bound genes between WT and Mut was performed using the DAVID tool [57].

### Metagene profiles

Previously published high-confidence human branch points were taken [58] and windows of 100 bp (–50 to +50) were extracted around the 5′ss, 3′ss and branch points for protein-coding genes. Strand-specific average profiles around 5′ss, 3′ss and branch points were created using the genomation Bioconductor package (version 1.24.0) [59].

### Custom multimapping approach

Barcodes and adapters were removed as described in previous section. PCR duplicates were removed using Clumpify from BBMAP (https://sourceforge.net/projects/bbmap) from the iCLIP reads before mapping to the GENCODE v30 (GRCh38.p12) using Bowtie 1.2.3 allowing all valid aligned reads (which is the multimapping step; Bowtie parameters: −v 2 −a). Individual replicates were merged, sorted and indexed using Sambamba [60]. PureCLIP [61] was used to detect the position with significant crosslink signal at snRNAs based on Hidden Markov model and snRNAs were visualized with the UCSC genome browser. Moreover, aligned reads (BAM file) were counted using script htseq-count [47] in GENCODE v30 annotation (gtf file) for each feature and features with a minimum read count of 10 were selected and normalized to the length of the feature.

## Results

### RP mutations in SNRNP200 alter its binding to U4 and U6 snRNAs

To understand how the S1087L and R1090L mutations affect the function of SNRNP200, we determined their RNA interaction profiles by iCLIP. We employed HeLa cells stably expressing either WT or RP mutants (S1087L or R1090L) fused to a GFP-containing LAP tag [42]. Proteins were expressed from bacterial artificial chromosomes (BACs) stably integrated into the genome. Expression from BACs resembles that of the corresponding endogenous genes and enables a direct comparison between WT and RP mutants. RP mutants were expressed at moderately lower levels than SNRNP200-WT, but the amount of isolated protein-RNA complexes was similar for WT and RP mutants, and iCLIP libraries were more complex in the case of SNRNP200-RP mutants (see below and Fig. S1) [42]. We used UV light to crosslink proteins to RNA, immunoprecipitated the protein-RNA adducts using anti-GFP antibodies under stringent conditions and identified crosslink events by deep sequencing as described previously [62–64] (Fig. S1B, C). Although helicases are generally difficult to crosslink due to their transient interactions and rapid translocations along an RNA substrate [65], we successfully prepared iCLIP libraries from all three SNRNP200-GFP cell lines (Fig. S1B, C). iCLIP reads were processed according to [64] and mapped to the human genome.

**Fig. 1:**
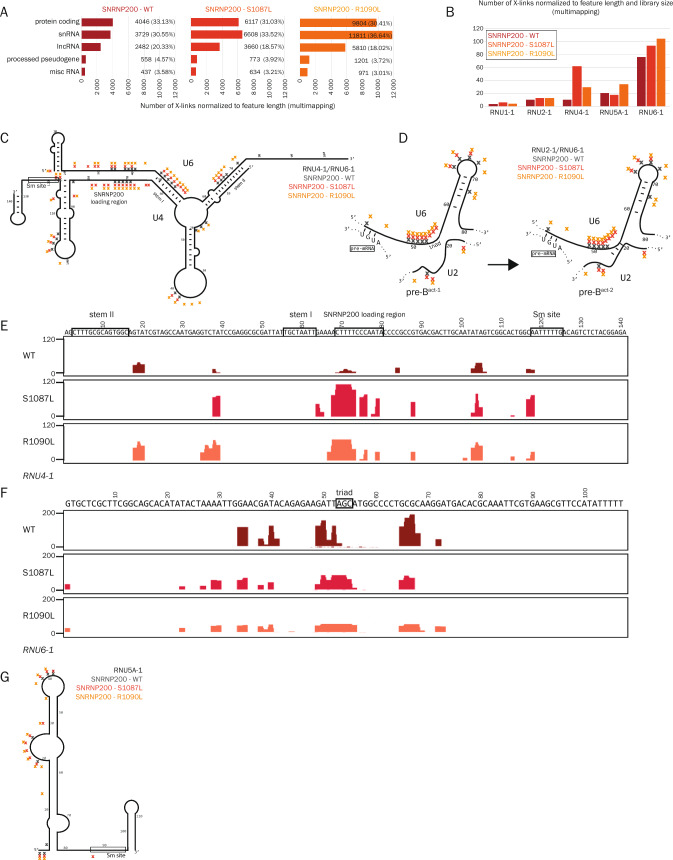
RP mutations S1087L and R1090L reduce the capacity of SNRNP200 to remodel the U4/U6 helix during splicing and compromise complex B activation. **A** Binding to the top five RNA biotypes is represented as number of crosslinks normalized to the feature length and also shown as a percentage for SNRNP200 WT, S1087L and R1090L variants. A custom multimapping approach was applied. **B** Number of crosslinks in individual snRNAs for SNRNP200 WT, S1087L and R1090L variants normalized to the gene length and to the size of the libraries. Schemes with indicated crosslinks of SNRNP200 (WT and RP variants) in U4/U6 snRNAs (**C**), and pre-B^act-1^ and pre-B^act-2^ complexes (the scheme of U2/U6 was adapted from [79]) (**D**). Crosslink profiles within *RNU4-1* (**E**) and *RNU6-1* (**F**); profiles are normalized to the size of the libraries. **G** Crosslinks of SNRNP200 (WT and RP variants) in U5 snRNA. The following color code is used for clarity: dark red (or gray in **C**, **D**, and **G**) - WT, red - S1087L and orange - R1090L

Since the main function of SNRNP200 is to unwind snRNAs, we applied a custom multimapping approach (see Materials and Methods) to obtain the best possible coverage on snRNAs, which are encoded by multiple copies of highly similar snRNA genes. The total number of crosslink events normalized to feature length was higher for both mutants compared to WT (Fig. 1A; Fig. S1D). Nearly one-third of WT SNRNP200 crosslink events mapped to snRNAs (30.6%), and this number further increased for S1087L (33.5%) and R1090L (36.6%) mutants. Among individual snRNAs, U4 showed the highest increase of crosslinks with RP variants, especially S1087L (Fig. 1B). Analysis of crosslinks at the individual-nucleotide level identified several U4 regions contacting SNRNP200 (Fig. 1C, E). We identified a new SNRNP200-U4 interaction site around nucleotides 103-106, which were previously suggested to be in close contact with SNRNP200 within the pre-catalytic B-complex (PDB: 5o9z, [66]). We further observed increased crosslinks of both RP variants within the U4 region downstream of the U4/U6 stem I. This is the loading site where SNRNP200 binds U4 to initiate stem I unwinding [67]. Accordingly, we detected crosslinks of both mutants to the region of U6 located upstream of stem I and opposite of the loading site on U4 (nucleotides 23-30) (Fig. 1C, F). Furthermore, crosslinks were more abundant for both mutants within nucleotides 35-39 of the first U4 loop, located between stems I and II of the U4/U6 duplex. The higher number of crosslinks reflects a stronger or longer binding of S1087L and R1090L variants to this specific U4 region. There are two scenarios compatible with this result. The mutations either increase SNRNP200 binding to U4 loading region, which can lead to more effective U4/U6 unwinding or contrary the mutations impair SNRNP200 helicase activity, translocation along U4 and unwinding of the U4/U6 duplex, which results in higher occupation of inactive SNRNP200 on U4 snRNA.

**Fig. 2:**
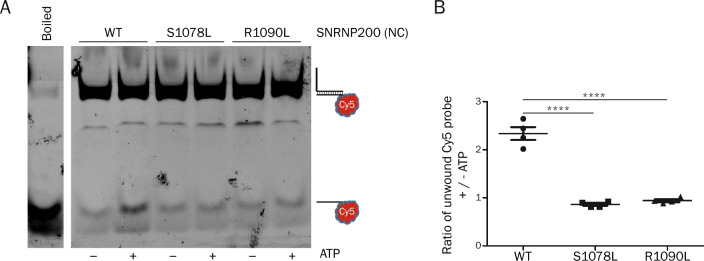
Helicase in vitro functional assay. **A** An RNA duplex was incubated with the purified N-terminal cassette (NC) of SNRNP200 (WT, S1087L and R1090L) with or without ATP and resolved in a native polyacrylamide gel. The boiled sample served as a positive control and was compiled from the same gel. **B** The ratio of unwound Cy5-labeled RNA oligo with/without addition of ATP. Statistical significance was assessed using a two-tailed unpaired *t*-test (**** indicates *p* ≤ 0.0001, *n* = 4)

To distinguish between these two alternatives, we directly tested SNRNP200 enzymatic activity by an in vitro helicase assay. To this end, we heterologously expressed and purified the N-terminal cassette of wild-type SNRNP200 that possesses an intrinsic helicase activity [43] together with both RP mutations. Recombinant enzymes were incubated with a fluorescently labeled model RNA duplex containing the U4 SNRNP200 loading region. Upon ATP addition, we observed RNA unwinding only for the WT protein, while the helicase activity was abolished for either of the two mutants (Fig. 2A, B). These findings are consistent with the model that increased iCLIP signal is caused by RP variants that are able to bind to the U4 loading region but are unable to translocate along the U4 snRNA and unwind the U4/U6 helix. In summary, our data show that RP mutations impair unwinding of the U4/U6 duplex both in vitro and in cellulo in the context of the spliceosome.

In addition to crosslinks to U6 snRNA single-stranded regions, SNRNP200 crosslinked abundantly to the U6 sequences that base pair with U4 (nucleotides 49-56 and 65-68). Because of the base pairing with U4 snRNA, these nucleotides are not accessible for crosslinking until U4/U6 is unwound during spliceosomal activation (UV crosslinking is inefficient in double-stranded RNA regions [68]). Thus, we suggest that SNRNP200 interacts with these nucleotides after remodeling of the U4/U6 duplex and formation of the (pre-)active spliceosome when both these regions are single-stranded (Fig. 1D). Consistent with this model, SNRNP200 crosslinked to nucleotides of U2 snRNA located in close proximity to the U6 A_53_G_54_C_55_ triad (Fig. S1E). Strikingly, while WT SNRNP200 crosslinked to the nucleotides G_49_A_50_T_51_T_52_ of U6 snRNA, both S1087L and R1090L variants crosslinked to an extended region encompassing the AGC triad, which should normally base pair with U2 snRNA upon transition from pre-B^act-1^ to pre-B^act-2^. The accessibility of these nucleotides for crosslinking indicates that the RP mutants might impair this transition (Fig. 1D). Within U5 snRNA, both SNRNP200 mutants showed extensive crosslinking to single-stranded regions, especially within the loop downstream of nt 20 (Fig. 1G). Altogether, these data suggest that RP mutations reduce SNRNP200’s helicase activity, which affects initial spliceosome activation as well as transition from pre-B^act-1^ to pre-B^act-2^.

The iCLIP experiment also showed a significant number of crosslinks of SNRNP200 to protein-coding genes (Fig. S2A). We analyzed protein-coding genes differentially bound by S1078L and R1090L mutants. Gene ontology (GO) analysis revealed that these genes are mostly involved in regulation of transcription by RNA polymerase II, protein phosphorylation and cytoskeleton organization. Next, we overlapped differently bound protein-coding genes with genes listed in the RetNet database (retnet.org), which contains the genes that most commonly cause retinal diseases (Fig. S2B). Interestingly, there are 25 genes from the RetNet database that are differentially bound by S1087L or R1090L variants and nine of these genes are directly linked with RP (Fig. S2B).

### RP mutations F2314L and Y2334N in PRPF8 do not affect its interaction with snRNAs

Next, we turned our attention to PRPF8 and applied the same iCLIP approach to determine whether RP mutations F2314L and Y2334N in PRPF8 affect its RNA-binding properties. We utilized HeLa cell lines stably expressing GFP-tagged WT or mutant PRPF8, which we characterized in detail previously [26]. We successfully prepared iCLIP libraries from all three cell lines (Fig. S3A, B). Both RP mutants crosslinked to RNA approximately 2.5-fold less efficiently than the WT protein (Fig. 2A, B), which might be partially due to the lower expression of both RP variants in comparison to WT PRPF8-GFP (Fig. S3C).

**Fig. 3:**
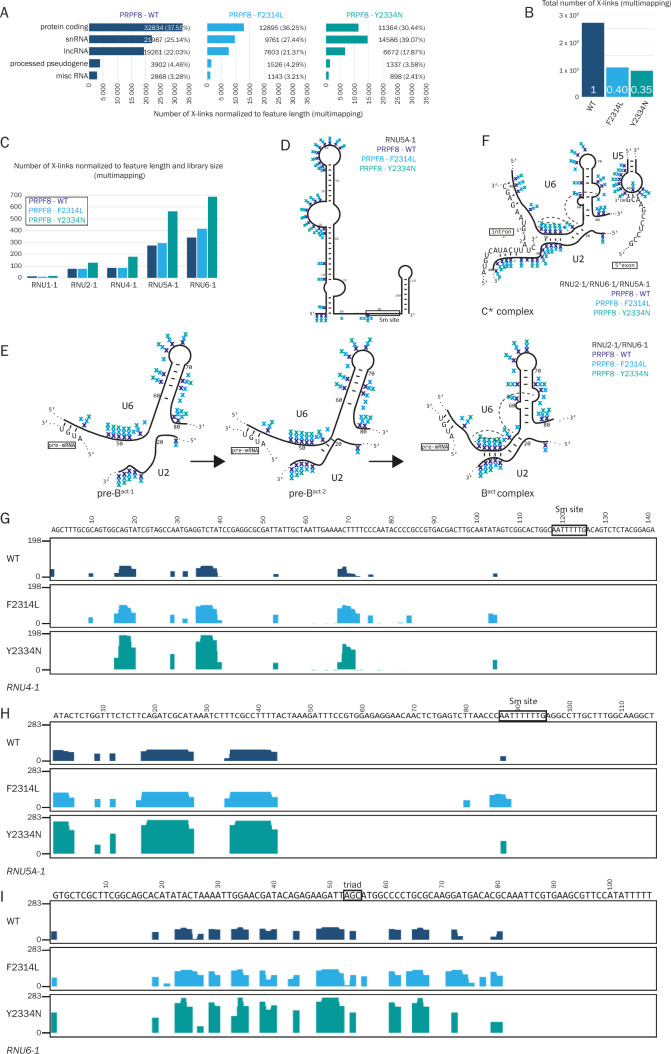
RP mutations F2314L and Y2334N do not alter the interaction of PRPF8 with snRNAs. **A** Binding to the top five RNA biotypes represented as number of crosslinks normalized to feature length and also shown as a percentage for PRPF8 WT, F2314L and Y2334N variants. A custom multimapping approach was applied. **B** Total number of crosslinks in PRPF8 (WT and RP variants) obtained using a multimapping approach. The size factor of the libraries is specified (white numbers) and further used for normalization in this figure. **C** Number of crosslinks in individual snRNAs for PRPF8 WT, F2314L and Y2334N variants; numbers are normalized to gene length and size of the libraries. Schemes with indicated crosslinks of PRPF8 (WT, F2314L and Y2334N variants) in U5 snRNA (**D**), U2 and U6 snRNAs in pre-B^act-1^, pre-B^act-2^ and B^act^ complexes (**E**) and the C* complex (**F**). Crosslink profiles normalized to the size of the libraries within *RNU4-1* (**G**), *RNU5A-1* (**H**) and *RNU6-1* (**I**). The following color code is used for clarity: dark blue - WT, azure blue - F2314L and turquoise blue - Y2334N

Similar to SNRNP200, PRPF8 is a component of the U5 snRNP and U4/U6⋅U5 tri-snRNP and contacts snRNAs within snRNPs and the spliceosome. To assess whether the interaction with snRNAs is altered by F2314L and Y2334N mutations, we applied a custom multimapping approach as described above. Compared to WT PRPF8, the Y2334N mutant crosslinked globally more to snRNAs (39% vs. 25%) and less to protein-coding genes (30% vs. 37%) and long non-coding RNAs (lncRNAs, 18% vs. 22%) (Fig. 3A). To compare iCLIP signals, we normalized to the total number of crosslinks (Fig. 3B) and analyzed binding of PRPF8 proteins to individual snRNAs (Fig. 3C). We observed a higher number of crosslinks for the Y2334N variant to all individual snRNAs. However, since there was a concomitant reduction in crosslinks to protein-coding genes and lncRNAs, we interpret these data to indicate that the increased binding of mutant PRPF8 to snRNAs is only relative. Furthermore, there were no qualitative differences in crosslink positions between RP mutants and WT PRPF8, further supporting the conclusion that RP mutations do not affect PRPF8-snRNA interaction (Fig. 3D–I). One minor difference was a broader interaction footprint of the F2314L variant on U6 between nucleotides 71 and 77. These nucleotides are single-stranded in the tri-snRNP and our finding may indicate some distortion of the tri-snRNP structure. In general, however, our iCLIP results do not suggest a major alteration of PRPF8’s interaction with snRNAs.

### PRPF8 F2314L and Y2334N mutations affect pre-mRNA binding efficiency without altering binding sites or splicing kinetics

**Fig. 4:**
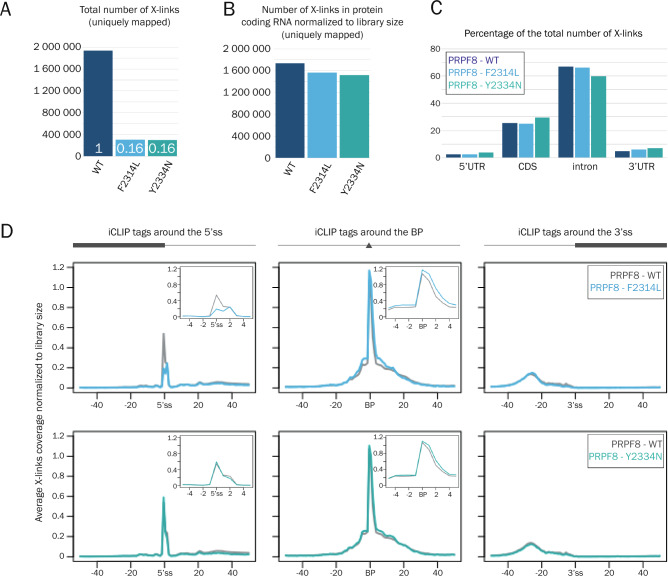
PRPF8 F2314L and Y2334N mutants show decreased binding to pre-mRNAs. **A** Total number of crosslinks uniquely mapped to PRPF8 (WT and RP variants). The size factor of the libraries is specified (white numbers) and further used for normalization in this figure. **B** Total number of crosslinks in protein-coding RNA for PRPF8 (WT and RP variants) normalized to the size of the libraries. **C** Proportional crosslinks mapped to individual features of protein-coding RNA (5′UTRs, coding sequences (CDS), introns, 3′ UTRs) in percentage for PRPF8 WT and RP variants. **D** Metaprofiles of PRPF8 WT, F2314L and Y2334N crosslinking. Values are shown as average crosslink coverage normalized to the size of the libraries. Above is depicted a pre-mRNA showing exons (rectangles), introns (line) and branch points (triangle)

In contrast to SNRNP200, PRPF8 is known to bind to pre-mRNA at multiple positions crucial for pre-mRNA splicing throughout the splicing reaction. Therefore, we compared the binding of WT PRPF8 and RP variants to pre-mRNAs by counting uniquely mapped reads only. Interestingly, both mutants crosslinked nearly 6.4-times less to RNA (304,127 crosslinks for F2314N, 301,029 for Y2334N and 1,939,474 for WT PRPF8, sum of three replicates). This may suggest that both RP mutants bind less efficiently to pre-mRNAs (Fig. 4A). To detect potential differences in the binding profiles, we normalized iCLIP signals to the size of the libraries and used the normalized data for further analyses (Fig. 4B). The majority of PRPF8 crosslinks were in intronic regions (over 67% of crosslinks in the case of WT PRPF8), whereas crosslinks within coding sequences (CDS) accounted only for 25% of total crosslinks within protein-coding RNA and a small percentage of crosslinks was identified in UTRs (2.5% in 5′ UTRs and 4.9% in 3′ UTRs in the case of PRPF8 WT). The percentage distribution of crosslinks within transcript features of both RP variants followed the same trend (Fig. 4C). These data are consistent with a predominant role of PRPF8 in splicing.

To determine whether RP mutations distort the binding profile of PRPF8 within introns, we analyzed metaprofiles of averaged crosslink coverage around 5′ss, BP and 3′ss. The results revealed sharp peaks at the 5′ss and BP and a smaller and wider peak 30 nts upstream of the 3′ss (Fig. 4D). These data are fully consistent with the well-described role of PRPF8 during splicing, and crosslinked regions either represent genuine PRPF8 binding or originate from the three-way junction of intron lariats still bound by PRPF8 as suggested previously [69]. Both RP mutants showed similar binding profiles as WT PRPF8. The only difference was a two-nucleotide shift of the peak at 5′ss in the case of the F2314L mutant, which might indicate a partial structural distortion of the spliceosome (Fig. 4D). These results suggest that RP mutations do not significantly alter the RNA-binding of PRPF8 to pre-mRNA intronic regions critical for splicing.

**Fig. 5:**
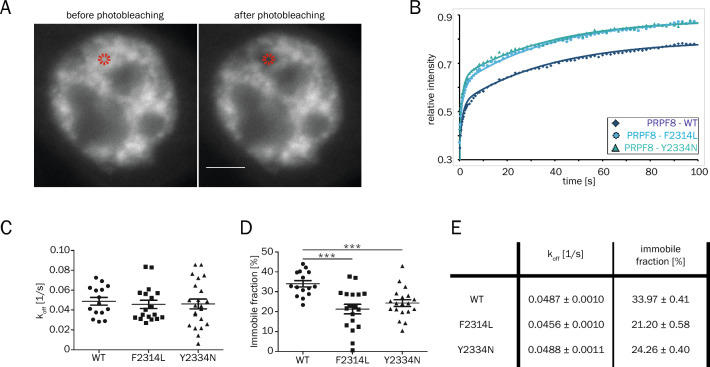
PRPF8 F2314L and Y2334N mutants show similar nuclear mobilities but distinct immobile fractions compared to WT PRPF8. **A** HeLa cell expressing WT PRPF8-GFP. Photobleaching was performed using a 488 nm LASER in a circular spot of 1.5 µm in diameter (labeled with a red circle) within the nucleoplasm. The cell is shown before and after photobleaching. Scale bar - 5 µm. **B** The fluorescence recovery was measured over the subsequent 100 s. Individual curves representing an average of 12-22 measurements (dots) were fitted bi-exponentially (solid lines). **C** Dissociation constants (*k*_off_) were obtained after fitting with the full model. **D** Immobile fractions determined after bi-exponential fitting. Statistical significance was assessed using a two-tailed unpaired *t*-test (*** *p* ≤ 0.001). **E** Mean values of *k*_off_ and percentage values of immobile fractions including SEM

To further address whether these mutations alter the behavior of PRPF8, we studied their interaction with pre-mRNAs in cellulo by FRAP as a proxy for splicing kinetics as previously established in our lab [52]. Here, we performed FRAP measurements in the nucleoplasm of HeLa cells expressing WT PRPF8-GFP and PRPF8 RP mutants. The GFP signal was bleached within a circular spot in the nucleoplasm, where the majority of pre-mRNA splicing takes place (Fig. 5A, B). Molecular dynamics inside nuclei are driven by diffusion and by interaction of the molecules with the nuclear environment [70]. FRAP recovery curves were fitted with the “full model” that determines diffusion rate and binding constants of the measured protein [71]. To reduce the number of fitted parameters, we utilized a PRPF8-GFP diffusion coefficient of 0.52 μm^2^/s previously determined by fluorescence correlation spectroscopy [52]. We fitted only the apparent binding constant *k*_on_* and the dissociation constant *k*_off_. The reverse value of *k*_off_ also reflects the binding time. *k*_off_ values obtained from FRAP measurement in the nucleoplasm were comparable for WT and both RP mutants (Fig. 5C, E). This indicates that RP mutants stay associated with pre-mRNAs for a similar time period as WT PRPF8. However, the course of the FRAP curves differed for WT and RP mutants; both mutant proteins exhibited a higher recovery than WT PRPF8 (Fig. 5B). To quantify the immobile fractions, we fitted the FRAP curves using a bi-exponential equation (see “Material and methods” section), which was more appropriate for the initial course of the curve. The immobile fractions were significantly smaller for F2314L and Y2334N in comparison to WT (Fig. 5D, E), which suggests that a lower number of mutated proteins interacts productively with pre-mRNAs. FRAP data are consistent with iCLIP results where we observed significantly fewer crosslinks for both RP variants. Being aware of the lower RP variant expression and semi-quantitative nature of the iCLIP data, we speculate that aberrant PRPF8 proteins enter splicing-competent spliceosomes less frequently than WT proteins. However, the similar *k*_off_ constant and binding profiles around splice sites indicate that once the mutated proteins associate with the spliceosome, they catalyze splicing with similar kinetics as WT PRPF8.

### The Y2334N mutation decreases pre-mRNA binding but does not affect splicing kinetics in RPE-1 cells

The expression of PRPF8 variants from BACs in HeLa cells might not reflect the autosomal-dominant inheritance optimally. Therefore, we used the CRISPR/Cas9 gene-editing technology to express the Y2334N mutation endogenously in the diploid hTERT immortalized RPE-1 cell line. We either tagged endogenous *PRPF8* with GFP (WT) as a control or introduced the Y2334N mutation together with the GFP tag into one *PRPF8* allele [37]. We tagged the C-terminus of PRPF8 (similarly to HeLa cells) because N-terminally tagged PRPF8 aberrantly localizes to the cytoplasm (Fig. S4). We first prepared iCLIP samples from RPE cell lines where only one allele had been edited to mimic the dominant inheritance in human RP. However, protein-RNA crosslinking was relatively inefficient in RPE cells and we did not obtain adequate coverage to properly evaluate the data. In this context it should be also noted that RP-associated mutations in the *Prpf8* gene provoked neurodegeneration only after mutagenesis of both alleles [37]. We therefore decided to edit both alleles and established cell lines expressing either PRPF8^WT^-GFP (termed RPE^WT^) or PRPF8^Y2334N^-GFP (termed RPE^Y2334N^), which were used in all subsequent experiments.

**Fig. 6:**
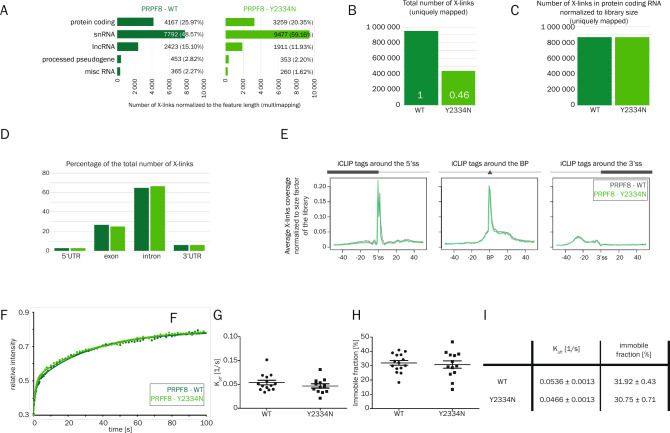
The Y2334N mutation decreases pre-mRNA binding but does not affect splicing kinetics in RPE cells. **A** Binding to the top five RNA biotypes represented as a number of crosslinks normalized to the feature length and also shown as a percentage for PRPF8 WT and Y2334N variant. A custom multimapping approach was applied. **B** Total number of crosslinks uniquely mapped for PRPF8 (WT and Y2334N). The size factor of the library is specified (white numbers) and further used for normalization in this figure. **C** Total number of crosslinks of PRPF8 (WT and Y2334N) in protein-coding RNA normalized to the size of the libraries. **D** Proportional crosslinks mapped to individual features of protein-coding RNAs (5′ UTR, exons, introns, 3′ UTR) in percentage for PRPF8 WT and Y2334N variant. **E** Metaprofiles of PRPF8 WT and Y2334N crosslinking around splice sites and branch points. Values are shown as average crosslink coverage normalized to the size of the library. The figure is accompanied by a pre-mRNA scheme showing exons (rectangle), introns (lines) and the branch points (triangle). **F** GFP fluorescence was bleached in the nucleoplasm and the recovery was monitored over 100 s. Average curve (dots) and bi-exponential fill (solid lines) are shown. **G** Dissociation constant (*k*_off_) and **H** percentage values of immobile fraction were comparable for WT and Y2334N. **I** *k*_off_ and percentage of immobile fraction including SEM

Our custom multimapping approach revealed that most crosslinks were found within snRNAs (over 48% in RPE^WT^ and 59% in RPE^Y2334N^) (Fig. 6A). Similar to HeLa cells, we did not observe any qualitative changes in PRPF8 interaction with snRNAs between WT and Y2334N PRPF8 (data not shown) and Y2334N PRPF8 crosslinked proportionally less to protein-coding RNAs than WT PRPF8. To study the interaction of PRPF8 with protein-coding RNAs, we analyzed uniquely mapped reads. Similar to HeLa cells, WT PRPF8 crosslinked to RNA more efficiently (948,944 crosslinks in total) than Y2334N PRPF8 (440,585 crosslinks in total) (Fig. 6B). After normalization to the size of the libraries, the difference in the number of crosslinks within protein-coding RNAs was negligible (Fig. 6C) and the crosslink distribution within pre-mRNA features was very similar for WT and Y2334N, with a majority of crosslinks found within intronic sequences (Fig. 6D). Metagene profiles of PRPF8^WT^ and PRPF8^Y2334N^ in RPE cells revealed an enrichment of crosslinks at the same positions around splice sites as in HeLa cells (Fig. 6E). Again, we observed distinct peaks at 5′ss and BP and a moderate peak upstream of 3′ss and PRPF8^WT^ and PRPF8^Y2334N^ binding to splice sites was almost identical. These results are in line with our findings from HeLa cells and show that the Y2334N mutation reduces binding to pre-mRNAs but does not alter the sites of interaction.

We further investigated the behavior of the Y2334N variant in RPE-1 cells with two copies of the Y2334N mutation and no endogenous WT PRPF8 by FRAP. To our surprise and in contrast to HeLa cells, that express endogenous WT PRPF8, FRAP measurements in the nucleoplasm of RPE cells (Fig. 6F) and consequent fitting of FRAP curves revealed similar immobile fractions for WT and Y2334N PRPF8 (Fig. 6H, I, compare with Fig. 5D, E). This suggests that the Y2334N variant is fully incorporated into snRNPs and the spliceosome in RPE-1 cells, where it does not compete with endogenous WT PRPF8. We observed a slightly lower *k*_off_ rate for the Y2334N mutant (Fig. 6G, I), indicating that PRPF8^Y2334N^ remains bound to pre-mRNAs for a longer time. Based on this finding we speculate that the RP mutation slows down splicing kinetics and/or release of mutated PRPF8 from the post-spliceosome complex. However, it should be noted that the variance of individual *k*_off_ measurements was high and the observed difference did not hold off a statistical test of significance. Together, these data suggest that the Y2334N mutation does not significantly change PRPF8’s interaction with pre-mRNAs and splicing kinetics in the RPE-1 cell line when two copies of the Y2334N variant and no endogenous WT PRPF8 is present.

### The Y2334N mutation alters the splicing and expression of selected genes

**Fig. 7:**
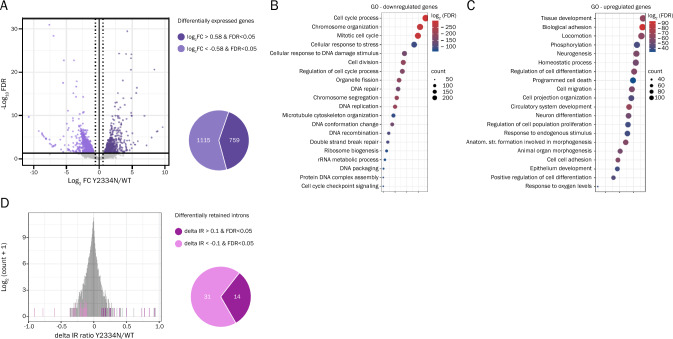
RP mutation Y2334N in PRPF8 alters gene expression in RPE cells. **A** Volcano plot of differential expression analysis. The criteria for differentially expressed genes were an FDR less than 0.05 combined with a minimum of 1.5-fold change in expression (|log_2_ FC| > 0.58). Pie chart shows proportion of genes downregulated (1115) and upregulated (759) in cells expressing the Y2334N mutant of PRPF8. Significantly enriched (FDR < 0.05) gene ontology (GO) biological process terms for genes that were downregulated (**B**) and upregulated (**C**) in RPE^Y2334N^ cells. Dot size represents the number of genes and dot color indicates significance. **D** Histogram showing the delta IR distribution. Differentially retained introns (|delta IR| > 0.1 & FDR < 0.05) are marked with colors, pie chart illustrates the proportion of introns more (31) and less retained (14) in the WT

Next, we analyzed whether the Y2334N substitution affects pre-mRNA splicing and mRNA expression using RNA-seq. Differential expression analysis using the DESeq2 method [48] revealed that 1874 out of 23,559 analyzed genes were differentially expressed. Approximately 60% of the genes were downregulated and 40% were upregulated in RPE^Y2334N^ cells (Fig. 7A). Six randomly selected genes (both up- and downregulated) were validated by RT-qPCR (Fig. S5A, B). We performed gene ontology (GO) enrichment analysis, and most GO terms among downregulated genes were related to cell cycle progression and processes associated with mitosis. Several GO terms were related to DNA damage and repair (Fig. 7B). The range of GO terms in the case of upregulated genes was broader (Fig. 7C); nevertheless, many processes closely related to the structure and function of the retina and/or RPE such as neurogenesis, neuron differentiation, cell-cell adhesion and epithelium development were enriched. To test whether down-regulated genes may result from nonsense-mediated decay (NMD) due to a lower splicing efficiency, we performed a knockdown of UPF1 or treated cells with cycloheximide (CHX) to inhibit NMD and analyzed three down-regulated genes by RT-qPCR (Fig. S5C). Neither 3 h of CHX treatment nor partial depletion of UPF1 (Fig. S5D) increased the levels of the tested genes, suggesting that NMD is not responsible for differential gene expression in the Y2334N mutant, at least in the case of the tested genes.

To assess whether PRPF8 mutant Y2334N might cause downregulation of gene expression through global reduction in splicing efficiency and intron retention, we used the IRFinder software [50]. To our surprise, we found only 45 differentially retained introns, of which two-thirds were actually more retained in RPE^WT^ cells (Fig. 7D). These 45 differentially retained introns are present in 41 genes of which 3 are downregulated and 6 upregulated in RPE^Y2334N^. Differentially retained introns in downregulated genes are longer with lower GC content compared to shorter, GC-richer introns from upregulated genes (Fig. S5E, F).

Analysis of a number of crosslinks of PRPF8 WT and the Y2334N variant revealed that they both interact with a significant proportion of protein-coding genes (Fig. 6A). Genes that are differentially bound by PRPF8 WT and Y2334N are mostly involved in cell adhesion, transcription and development of nervous system (Fig. S6A). We further overlapped these differentially bound genes with genes from the RetNet database. We observed 14 overlapped genes; four of them are causative for RP (Fig. S6B). Finally, we compared differentially bound genes with differentially expressed and spliced genes. GO analysis revealed similar GO terms as in Fig. S6A and interestingly, also a response to retinoic acid (Fig. S6C–E). These data suggest that the Y2334N mutation affects binding of PRPF8 to a specific group of genes and changes their expression.

Although we did not detect any significant changes in pre-mRNA binding and splicing kinetics, these data show that the Y2334N substitution modulates the behavior of PRPF8, which results in changes in splicing of selected genes and expression of hundreds of genes, some of which are involved in RPE structure and function and are associated with RP.

## Discussion

Mutations in PRPF8 and SNRNP200 as well as several other splicing factors cause retinal cell degeneration, leading to vision impairment and eventually to blindness. In the last two decades, intensive research on RP-linked mutations in splicing factors has revealed that most mutations result in reduced levels of splicing-competent snRNPs [26, 27, 30–32]. However, a few substitutions in SNRNP200 and PRPF8 do not have an apparent effect on tri-snRNP formation but still induce severe retinal degeneration with early onset [26, 40–42, 72, 73]. Some of these mutations were studied in vitro ignoring the complex spliceosomal context or in basic model organisms such as budding yeast. Here, we characterized four of these RP variants by iCLIP to thoroughly map their interactions with RNA genome-wide at nucleotide resolution within the context of the spliceosome. We further employed FRAP to monitor the behavior of aberrant PRPF8 in the cell nucleus.

Although SNRNP200 is an RNA helicase that contacts its substrate only transiently, we observed a clear interaction of SNRNP200 with several regions of U2, U4, U5 and U6 snRNAs. To the best of our knowledge, we present here the first successful iCLIP analysis of SNRNP200. Our data reveal multiple contacts between SNRNP200 and its substrates, including aberrant contacts in the case of mutants S1087L and R1090L. These mutations increase the footprint of the RNA-binding profile and appear to enhance the binding of SNRNP200 to both U4 and U6 upstream of the U4/U6 stem I. This is the region where SNRNP200 interacts with U4/U6 snRNAs prior to the unwinding of the U4/U6 duplex during catalytic activation of the spliceosome. This strongly indicates that those mutations result in the stalling of SNRNP200 on the U4/U6 snRNAs and impair its helicase activity in the context of the tri-snRNP. Both mutated amino acids in SNRNP200 are found in the RNA-binding pocket of the active N-terminal helicase cassette and might either strengthen its interaction with RNA, thereby slowing down its translocation along the RNA substrate, or directly reduce its helicase activity. These findings are consistent with data from in vitro experiments, which showed that RP variants in human and yeast SNRNP200 impaired U4/U6 unwinding in vitro [43, 72, 74]. Moreover we showed attenuated helicase activity for both S1087L and R1090L mutations here. However, our results do not support the suggestion that RP-mimicking mutations weaken SNRNP200 affinity for U4/U6 snRNAs [74]. On the contrary, we observe a stronger interaction of SNRNP200 with its U4 and U6 snRNAs. Together with our previous analyses of both RP mutants by FRAP, which revealed approximately two- to threefold shorter interaction times with RNA [42], we speculate that the reduced unwinding activity of SNRNP200 rather delays spliceosome activation and triggers the partial removal of stalled splicing complexes from pre-mRNAs.

SNRNP200 remains associated with the catalytically activated spliceosome and has been suggested to be required again during splicing catalysis and spliceosome disassembly [18, 75–77]. Accordingly, we detected abundant crosslinks of SNRNP200 within a region of U6 snRNA that base pairs with U4 snRNA and is thus inaccessible for UV-crosslinking while in the U4/U6 duplex. However, this U6 region is single-stranded following spliceosome activation. Within this region, WT SNRNP200 crosslinks are limited to nucleotides 49-52, immediately preceding the A_53_G_54_C_55_ triad, which is critical for splicing and base pairs with U2 snRNA upon transition from the pre-B^act^ to the B^act^ complex (51, 52). The extended crosslinking of mutants S1087L and R1090L to this triad strongly suggests that this transition is impaired in the presence of those mutants. Thus, our results indicate that SNRNP200 indeed plays additional roles during/following the activation of the spliceosome as suggested previously [18, 75–77].

Consistent with its central position in the spliceosome, we detected extensive interactions between PRPF8 and U2, U4, U5 and U6 snRNAs. However, in contrast to SNRNP200, we did not observe substantial changes in snRNA-binding profiles of PRPF8 mutants F2314L and Y2334N compared to WT PRPF8. Within pre-mRNA sequences, those mutants also did not display any marked differences in their binding profiles around motifs essential for splicing (5′ss, BP and 3′ss). However, results from our FRAP approach revealed that those RP mutations do not affect the time of interaction between PRPF8 and pre-mRNAs (i.e. *k*_off_ values) but do reduce their immobile fraction. Our interpretation is that a smaller fraction of PRPF8-containing tri-snRNPs present in the cell at a given time are being actively engaged in splicing, but those that are, perform splicing with normal kinetics. Consistent with this, FRAP analysis of RPE cells where both *PRPF8* alleles harbored the Y2334N mutation revealed both normal *k*_off_ values and immobile fractions compared to WT PRPF8. In HeLa cell model, tri-snRNPs containing RP variants are probably outcompeted by those containing endogenous PRPF8 for joining active spliceosomes. However, RPE^Y2334N^cells with no other options use tri-snRNPs containing mutated PRPF8 and execute splicing reactions “reasonably” well. Since the Y2334N mutation is inherited dominantly, it might be actually this competition of WT PRPF8 and Y2334N variant that drives the disease phenotype in retinal cells. Besides, RP caused by mutations in splicing factors is nonsyndromic, which indicates that defects induced by mutations are minimal and most human cells and tissues are able to tolerate them.

Nevertheless, we detected changes in the expression of hundreds of genes in RPE cells, many of which involved in cell-cell adhesion, epithelium development, neurogenesis or neuron differentiation, which might be involved in RP pathogenesis in patients. Indeed, 14 genes that are differentially bound by PRPF8^Y2334N^ in RPE cells have been associated with various retinal dystrophies and four genes (MVK, PDSS1, MERTK and SEMA4A) are involved in RP. We also observed the PRPF8 splicing autoregulation [78], which can induce perturbations in the splicing regulatory network. Analysis of intron retention events revealed surprisingly few changes, and in most cases introns were actually more retained in WT cells. This is consistent with previous observations of favored intron retention in control RPE compared to PRPF8^p.Val2325-Glu2331del^ RP patient cells [78]. The authors suggested that, in the context of an intron retention “program”, retained introns might have specific roles in normal physiology of RPE. Whether impaired retention of specific introns in the RPE cells of patients harboring this or any other RP mutation (including Y2334N) contributes to the pathology of the disease remains to be determined.

In this study, we combined iCLIP and FRAP to monitor how RP-linked mutations affect the activity of splicing factors in vivo. Our results provide clues as to how RP mutations alter the behavior of mutated SNRNP200 and PRPF8 and lead us to propose that changes in splicing and expression of genes important for retinal function are the main molecular mechanism that underlies retinal degeneration caused by mutations in splicing factors.

## Supplementary Information

Below is the link to the electronic supplementary material.

Supplementary file1 (PDF 834 KB)

## Data Availability

The raw data of the RNA-seq and iCLIP were deposited in the GEO database under accession numbers GSE213807 (Super Series), GSE213805 and GSE213806 (Subseries).
