# Supplementary material for: Retinitis pigmentosa-linked mutations impair the snRNA unwinding activity of SNRNP200 and reduce pre-mRNA binding of PRPF8
Source: Cell Mol Life Sci. 2025 Mar 5;82(1):103. doi: 10.1007/s00018-025-05621-z (PMC11883072; doi:10.1007/s00018-025-05621-z)
Supplement: Supplementary file 1 — Supplementary file1 (PDF 834 KB) [file 18_2025_5621_MOESM1_ESM.pdf]

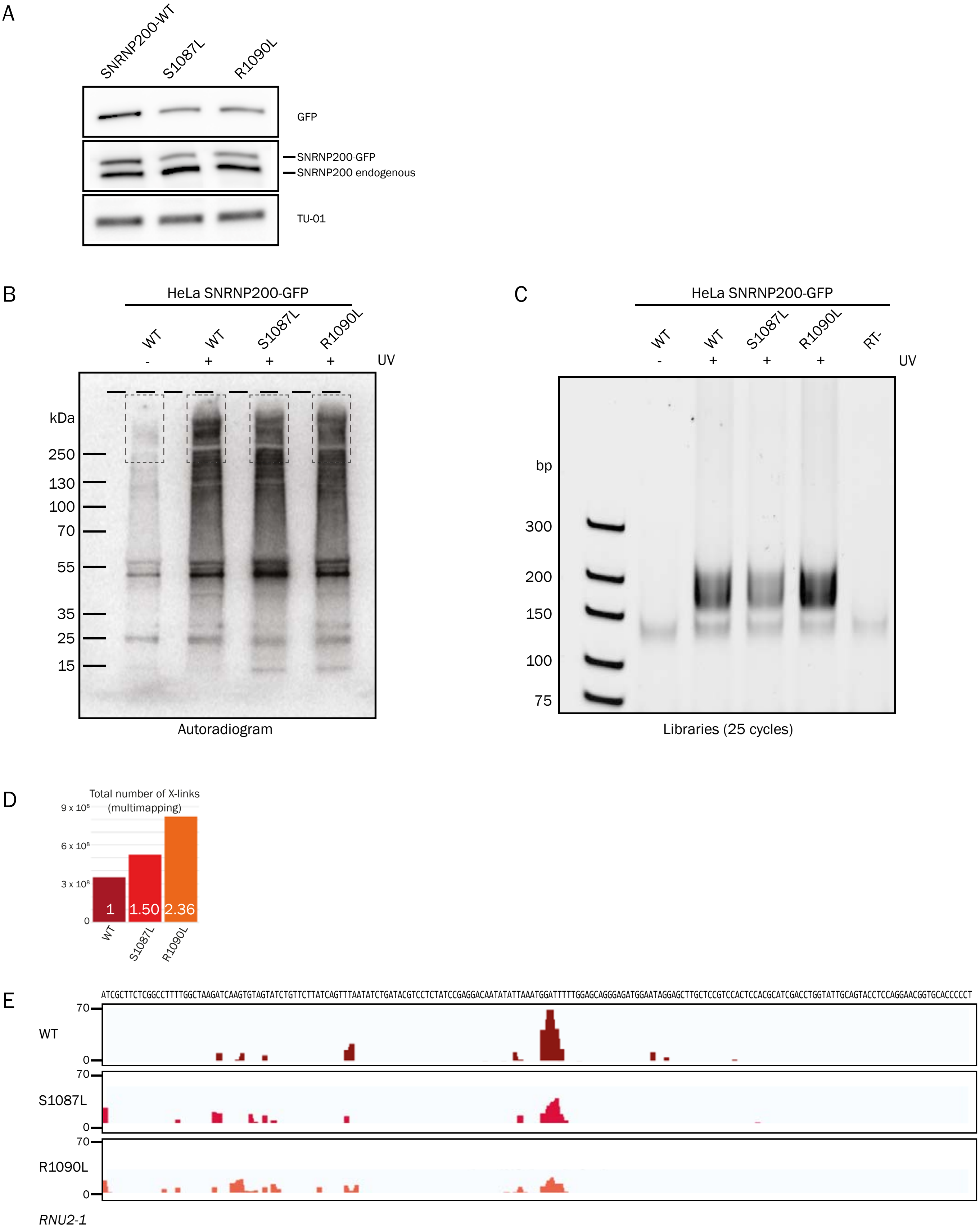
A
SNRNP200-WT
S1087L
R1090L
GFP
SNRNP200-GFP
SNRNP200 endogenous
TU-01
B
HeLa SNRNP200-GFP
WT
WT
S1087L
R1090L
- + + + UV
kDa
250
130
100
70
55
35
25
15
Autoradiogram
C
HeLa SNRNP200-GFP
WT
WT
S1087L
R1090L
RT-
- + + + UV
bp
300
200
150
100
75
Libraries (25 cycles)
D
Total number of X-links (multimapping)
9 x 10⁸
6 x 10⁸
3 x 10⁸
0
1
1.50
2.36
WT
S1087L
R1090L
E
ATCGCTTCTCGGCCTTTTGGCTAAGATCAAGTGTAGTATCTGTTCTTATCAGTTTAATATCTGATACGTCCTCTATCCGAGGACAATATATTAAATGGATTTTTGGAGCAGGGAGATGGAATAGGAGCTTGCTCCGTCCACTCCACGCATCGACCTGGTATTGCAGTACCTCCAGGAACGGTGCACCCCCT
70
0
WT
S1087L
R1090L
RNU2-1

Fig. S1

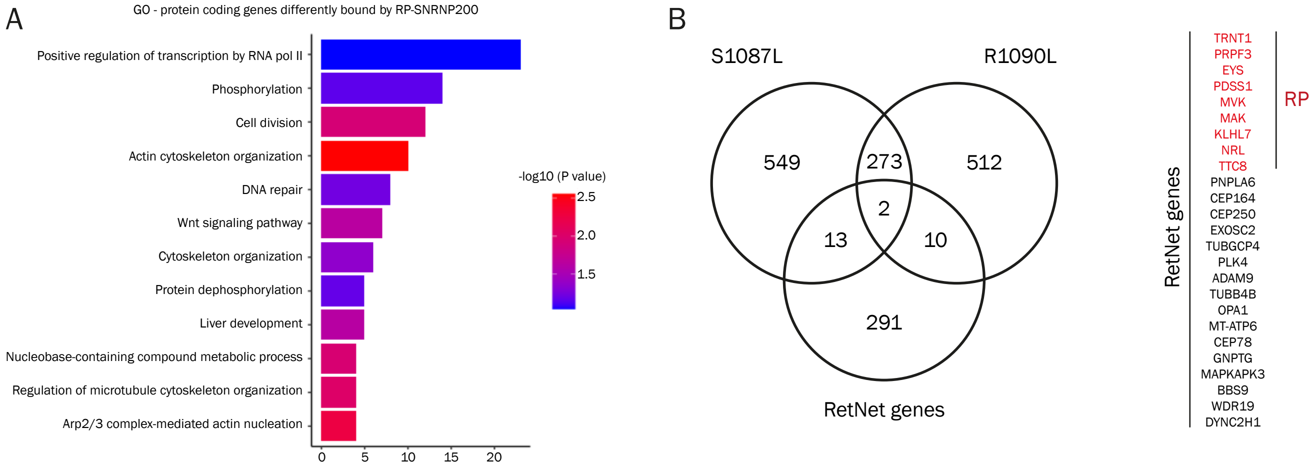
A
GO - protein coding genes differently bound by RP-SNRNP200
Positive regulation of transcription by RNA pol II
Phosphorylation
Cell division
Actin cytoskeleton organization
DNA repair
Wnt signaling pathway
Cytoskeleton organization
Protein dephosphorylation
Liver development
Nucleobase-containing compound metabolic process
Regulation of microtubule cytoskeleton organization
Arp2/3 complex-mediated actin nucleation
0
5
10
15
20
-log10 (P value)
2.5
2.0
1.5
B
S1087L
R1090L
549
273
512
2
13
10
291
RetNet genes
TRNT1
PRPF3
EYS
PDSS1
MVK
MAK
KLHL7
NRL
TTC8
RP
PNPLA6
CEP164
CEP250
EXOSC2
TUBGCP4
PLK4
ADAM9
TUBB4B
OPA1
MT-ATP6
CEP78
GNPTG
MAPKAPK3
BBS9
WDR19
DYNC2H1
RetNet genes

Fig. S2

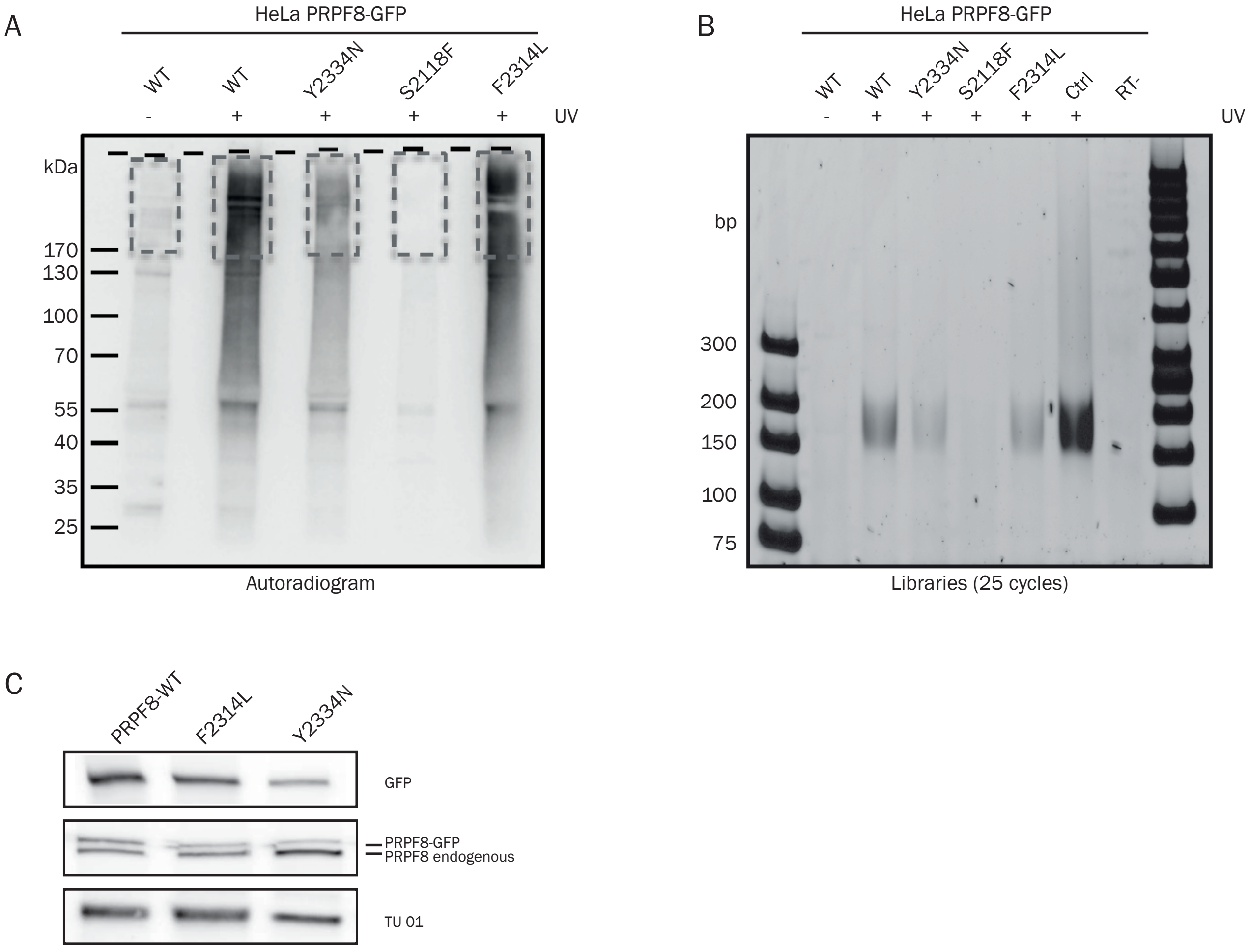
A
HeLa PRPF8-GFP
WT
WT
Y2334N
S2118F
F2314L
-
+
+
+
+
UV
kDa
170
130
100
70
55
40
35
25
Autoradiogram
B
HeLa PRPF8-GFP
WT
WT
Y2334N
S2118F
F2314L
Ctrl
RT-
-
+
+
+
+
+
UV
bp
300
200
150
100
75
Libraries (25 cycles)
C
PRPF8-WT
F2314L
Y2334N
GFP
PRPF8-GFP
PRPF8 endogenous
TU-01

Fig. S3

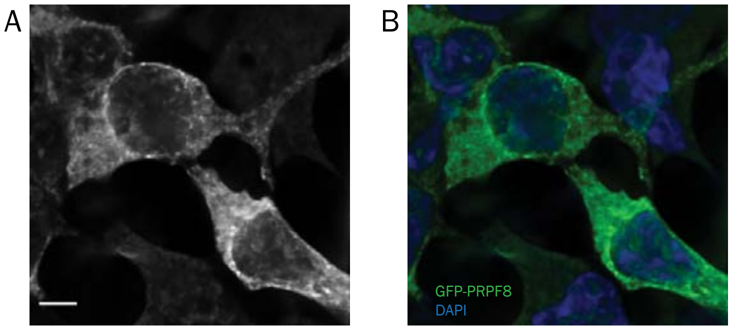

Fig. S4

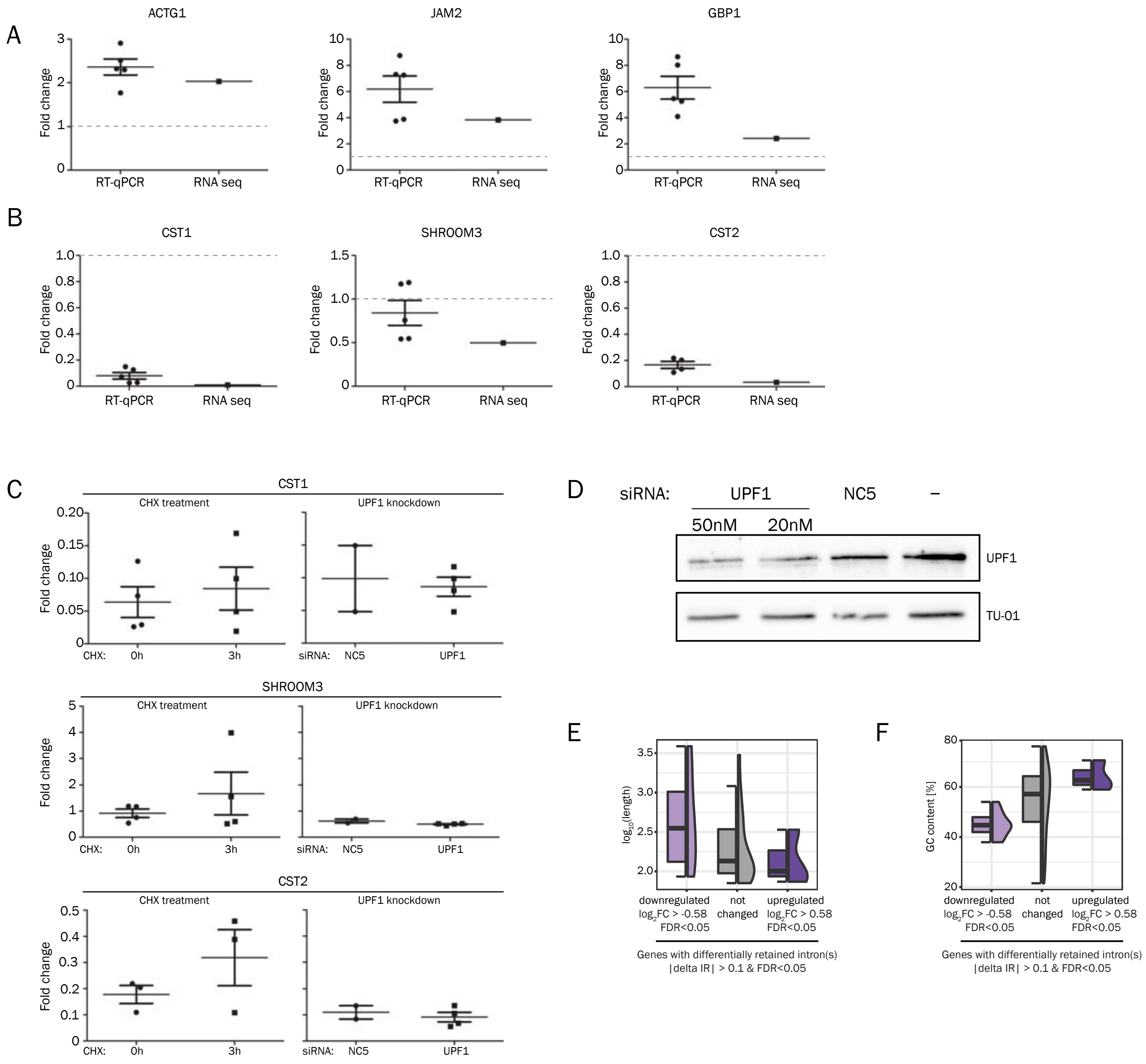

A
ACTG1
JAM2
GBP1
Fold change
RT-qPCR
RNA seq
B
CST1
SHROOM3
CST2
C
CHX treatment
UPF1 knockdown
CHX: 0h 3h
siRNA: NC5 UPF1
D
siRNA: UPF1 NC5 –
50nM 20nM
UPF1
TU-01
E
log10(length)
F
GC content [%]
downregulated log2FC > -0.58 FDR<0.05
not changed
upregulated log2FC > 0.58 FDR<0.05
Genes with differentially retained intron(s)
|delta IR| > 0.1 & FDR<0.05

Fig. S5

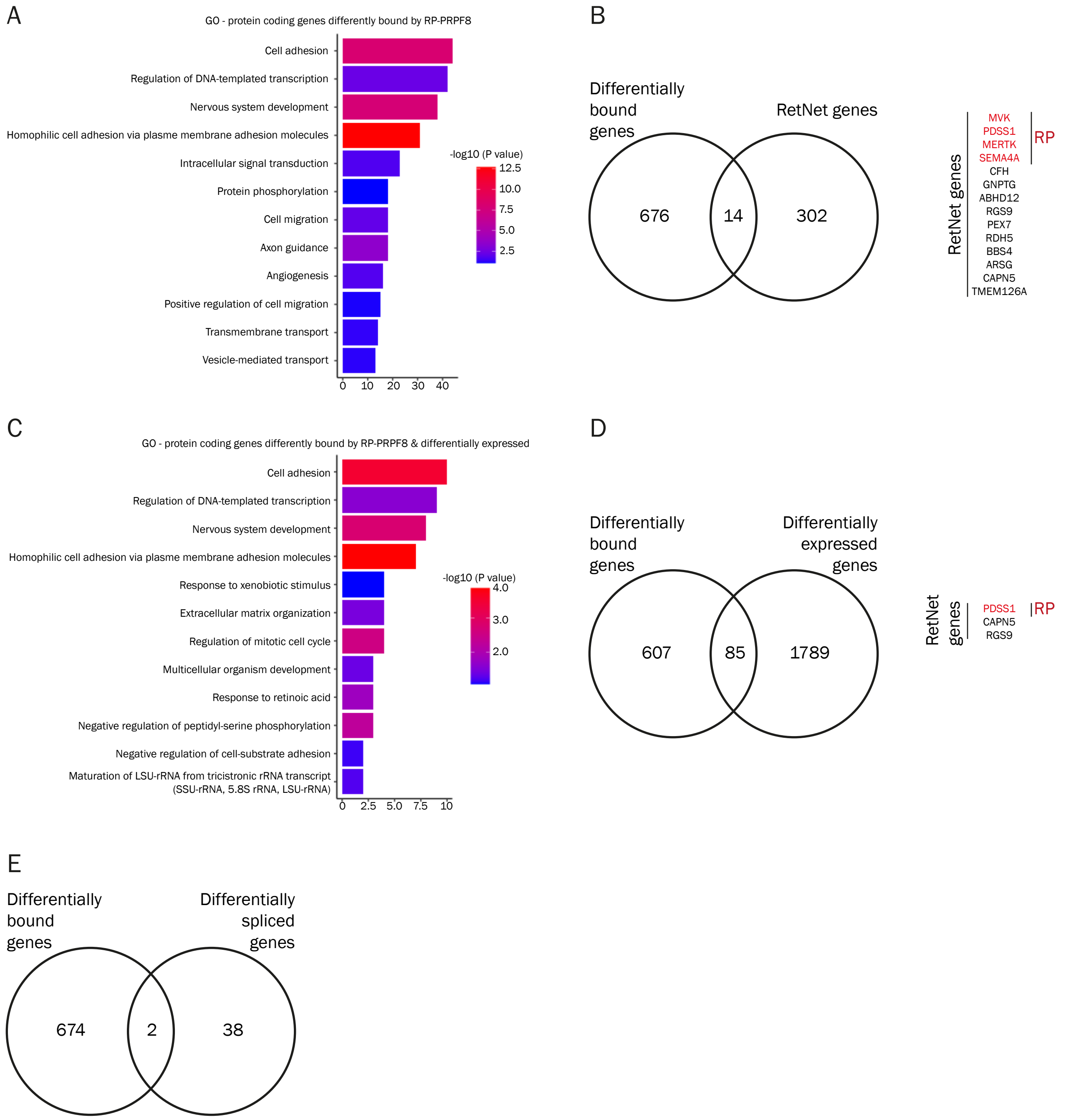

A
GO - protein coding genes differently bound by RP-PRPF8
Cell adhesion
Regulation of DNA-templated transcription
Nervous system development
Homophilic cell adhesion via plasme membrane adhesion molecules
Intracellular signal transduction
Protein phosphorylation
Cell migration
Axon guidance
Angiogenesis
Positive regulation of cell migration
Transmembrane transport
Vesicle-mediated transport
0 10 20 30 40
-log10 (P value)
12.5
10.0
7.5
5.0
2.5
B
Differentially bound genes
RetNet genes
676
14
302
RetNet genes
MVK
PDSS1
MERTK
SEMA4A
RP
CFH
GNPTG
ABHD12
RGS9
PEX7
RDH5
BBS4
ARSG
CAPN5
TMEM126A
C
GO - protein coding genes differently bound by RP-PRPF8 & differentially expressed
Cell adhesion
Regulation of DNA-templated transcription
Nervous system development
Homophilic cell adhesion via plasme membrane adhesion molecules
Response to xenobiotic stimulus
Extracellular matrix organization
Regulation of mitotic cell cycle
Multicellular organism development
Response to retinoic acid
Negative regulation of peptidyl-serine phosphorylation
Negative regulation of cell-substrate adhesion
Maturation of LSU-rRNA from tricistronic rRNA transcript (SSU-rRNA, 5.8S rRNA, LSU-rRNA)
0 2.5 5.0 7.5 10
-log10 (P value)
4.0
3.0
2.0
D
Differentially bound genes
Differentially expressed genes
607
85
1789
RetNet genes
PDSS1
RP
CAPN5
RGS9
E
Differentially bound genes
Differentially spliced genes
674
2
38

Fig. S6

Primers used for RT-qPCR validation of differential gene expression analysis

Name	Sequence
ACTG1_F	AGGCCAACAGAGAGAAGATG
ACTG1_R	CATGACAATGCCAGTGGTG
GBP1_F	GAGGCATCCAGGTCAACG
GBP1_R	CTGAGTTCTCTATCTGGGCC
SHROOM3_F	AGGATCCTACAAGACCCTCA
SHROOM3_R	TTAGAGGCACCAGTATCTGC
JAM2_F	AAGTTAGTGCCCCATCTGAG
JAM2_R	CTCAGAGCAGAAGAGGGTAC
CST2_F	CCCTTCACTTTGTCATCAGC
CST2_R	GAAGAAGTAATTCACCCCGC
CST1_F	GGTACTAAGAGCCAGGCAAC
CST1_R	GTTCTGGCTGTTCATGGAAG

Table S1

Supplementary files legend

Figure S1. SNRNP200 iCLIP: autoradiography and iCLIP libraries

(A) Western blot analysis shows the expression of GFP-tagged and endogenous SNRNP200 in all stable SNRNP200-GFP HeLa cell lines, tubulin (TU-01) is used as a loading control. (B) Autoradiogram of radioactively labeled SNRNP200-GFP:RNA complexes. immunoprecipitated with anti-GFP antibody after UV crosslinking in HeLa cells. Non-UV crosslinked cells are included as negative control (UV-). Areas in dotted rectangles indicate the region that was cut from the membrane for subsequent RNA isolation. (C) Final iCLIP library after size selection. A control without RT is included (RT-). (D) The total number of crosslinks mapped to SNRNP200 (WT, S1087L, R1090L) was obtained by the multimapping approach. The size factor of the library is specified (white numbers) for each SNRNP200 variant. (E) Crosslink profile within *RNU2-1* normalized to the size factor of the library.

Figure S2. Protein-coding genes differentially bound by SNRNP200

(A) Top 12 Gene Ontology (GO) categories enriched for differentially bound protein-coding genes between WT and mutant samples. The bar graph shows the number of genes supporting each category and the -log10(p-value) of enrichment. GO enrichment analysis was performed using the DAVID tool. (B) Venn diagrams showing the relation between protein-coding genes differentially bound by S1087L and R1090L of SNRNP200 and with genes listed in RetNet database. Genes from RetNet database that are differently bound by S1087L or R1090L and listed and those that cause of RP specifically highlighted.

Figure S3. PRPF8 expression and iCLIP: autoradiography and iCLIP libraries

(A) Autoradiogram of radioactively labeled PRPF8-GFP:RNA complexes. Note: S2118F is different RP-linked mutation, but its crosslinking was not effective and therefore has not been studied further. (B) Final iCLIP library after size selection. (C) Western blot analysis shows the intensity of GFP signals in all stable PRPF8-GFP HeLa cell lines, tubulin (TU-01) is used as a loading control.

Figure S4. N-terminally tagged PRPF8 aberrantly localizes to the cytoplasm

(A) Expression of PRPF8 protein (WT) tagged with GFP on its N terminus and (B) merged picture containing DAPI (DNA visualization). Scale bar - 5 µm.

Figure S5. Six selected genes that are differentially expressed based on RNAseq analysis were validated by RT-qPCR

(A) genes that are more expressed in WT (B) genes more expressed in Y2334N mutant. Fold change in expression is calculated as $2^{-[(\text{Ct gene in Y2334N} - \text{Ct reference in Y2334N}) - \text{Ct gene in WT} - \text{Ct reference in WT})]}$, GAPDH was used as a reference gene. Values calculated from RT-qPCR validation and values obtained from RNA seq are shown. (C) RT-qPCR analysis of down-regulated genes after NMD inhibition either by CHX treatment (3h) or knockdown of UPF1 (30nM siRNA targeting UPF1). (D) Knockdown of UPF1 in RPE cells; TU-01 was used as a loading control. NC5 is a negative control siRNA. (E) Differentially retained introns are longer in genes with downregulated expression and shorter in upregulated genes and at the same time (F) the GC content is higher in differentially retained introns in genes with upregulated expression.

Figure S6. Protein-coding genes differentially bound by PRPF8

(A) Top 12 Gene Ontology (GO) categories enriched for differentially bound protein-coding genes between PRPF8 WT and Y2334N variant. The bar graph shows the number of genes supporting each category and the -log10(p-value) of enrichment. GO enrichment analysis was performed using the DAVID tool. (B) Venn diagrams showing the relation between protein-coding genes differentially bound by Y2334N of PRPF8 and WT and with genes listed in RetNet database. Genes from RetNet database that are differently bound by Y2334N and listed and those that cause of RP specifically highlighted. (C) GO analysis of genes that are differentially bound by Y2334N and differentially expressed in the same time. (D) Venn diagram showing the relation between genes that are differentially bound by Y2334N with genes differentially expressed in $PRPF8^{Y2334N}$ in RPE cells. (E) Overlap between differentially bound and differentially spliced transcripts in RPE cells.

Table S1. List of primers used for RT-qPCR validation of differential gene expression analysis.
